# A gamma-distribution convolution model of ^99m^Tc-MIBI thyroid time-activity curves

**DOI:** 10.1186/s40658-016-0166-z

**Published:** 2016-12-16

**Authors:** Carl A. Wesolowski, Surajith N. Wanasundara, Michal J. Wesolowski, Belkis Erbas, Paul S. Babyn

**Affiliations:** 1 Department of Medical Imaging, Royal University Hospital, College of Medicine, University of Saskatchewan, 103 Hospital Drive, Saskatoon, SK S7N 0W8 Canada; 2 Nuclear Medicine, Department of Radiology, Faculty of Medicine, Memorial University of Newfoundland, 300 Prince Philip Drive, St. John’s, NL A1B 3X7 Canada; 3 Department of Nuclear Medicine, Hacettepe University Medical School, Sıhhiye, Ankara 06100 Turkey

**Keywords:** Gamma distribution convolution, ^99m^Tc-hexakis-methoxy-isobutyl-isonitrile, Thyroid, Time-activity curve, Gamma camera

## Abstract

**Background:**

The convolution approach to thyroid time-activity curve (TAC) data fitting with a gamma distribution convolution (GDC) TAC model following bolus intravenous injection is presented and applied to ^99m^Tc-MIBI data. The GDC model is a convolution of two gamma distribution functions that simultaneously models the distribution and washout kinetics of the radiotracer.

The GDC model was fitted to thyroid region of interest (ROI) TAC data from 1 min per frame ^99m^Tc-MIBI image series for 90 min; GDC models were generated for three patients having left and right thyroid lobe and total thyroid ROIs, and were contrasted with washout-only models, i.e., less complete models. GDC model accuracy was tested using 10 Monte Carlo simulations for each clinical ROI.

**Results:**

The nine clinical GDC models, obtained from least counting error of counting, exhibited corrected (for 6 parameters) fit errors ranging from 0.998% to 1.82%. The range of all thyroid mean residence times (MRTs) was 212 to 699 min, which from noise injected simulations of each case had an average coefficient of variation of 0.7% and a not statistically significant accuracy error of 0.5% (*p* = 0.5, 2-sample paired *t* test). The slowest MRT value (699 min) was from a single thyroid lobe with a tissue diagnosed parathyroid adenoma also seen on scanning as retained marker. The two total thyroid ROIs without substantial pathology had MRT values of 278 and 350 min overlapping a published ^99m^Tc-MIBI thyroid MRT value. One combined value and four unrelated washout-only models were tested and exhibited R-squared values for MRT with the GDC, i.e., a more complete concentration model, ranging from 0.0183 to 0.9395.

**Conclusions:**

The GDC models had a small enough TAC noise-image misregistration (0.8%) that they have a plausible use as simulations of thyroid activity for querying performance of other models such as washout models, for altered ROI size, noise, administered dose, and image framing rates. Indeed, of the four washout-only models tested, no single model approached the apparent accuracy of the GDC model using only 90 min of data. Ninety minutes is a long gamma-camera acquisition time for a patient, but a short a time for most kinetic models. Consequently, the results should be regarded as preliminary.

## Background

The use of technetium-99m hexakis-methoxy-isobutyl-isonitrile (^99m^Tc-MIBI) as a thyroid and parathyroid imaging agent was first proposed in the late 1980s [1]. Since its inception, ^99m^Tc-MIBI scintigraphy has been shown to be more accurate and sensitive than comparable imaging techniques and is currently used to detect and localize differentiated thyroid cancer [2, 3], parathyroid adenoma [1, 4], parathyroid hyperplasia [5, 6], and parathyroid carcinoma [4, 7].

Several ^99m^Tc-MIBI image acquisition protocols and analysis techniques have been devised over the last quarter century to aid in the detection of abnormal thyroid and parathyroid tissue, these include dual-phase scintigraphy [8], factor analysis of a dynamic scan [9], and time-activity curve (TAC) analysis of a dynamic scan [10]. TAC analysis provides a means of differentiating between normal and abnormal tissues by comparing of radiotracer uptake and washout in different regions of interest (ROIs). ^99m^Tc-MIBI does not undergo significant chemical changes in the body and therefore is passively taken up in the thyroid and parathyroid tissues and is cleared rapidly from the blood [11]. TAC analysis also has the potential to provide detailed pharmacokinetic information from ROIs. Pharmacokinetic parameters, such as the tracer mean residence time (MRT) and elimination half-life can be used to quantify and characterize disease states, which ultimately may aid in the diagnosis of thyroid and parathyroid disease.

It is a common practice to inject a bolus of a drug into a vein, but to take images or venous samples elsewhere. Before the time of first arrival of activity in the sampling region, there is no drug and no signal in that remote site. Most pharmacokinetic bolus models ignore this initial mixing and are washout-only models having a maximum amplitude initially. Thus, at the earliest times following a bolus, there is a severe mismatch between a washout model and the signal it models. Consequently, most of the routinely used pharmacokinetic models, e.g., classic compartmental washout models, do a poor job of fitting early-time organ activity [12, 13]. Thus, washout functions do not fit concentration or time-activity curves well enough to serve as accurate simulation study models. To model the entire TAC accurately, it is necessary to use a mathematical model that includes at least some of the drug arrival and organ drug distribution effects. Convolutions of two functions as an impulse response model can approximate the entire TAC accurately [14, 15]. Of these two functions, the first or fast function can be thought of as an organ feed or stimulus function consisting of rapidly changing blood pool background within the organ. The second can be thought of as the response or result of bathing the parenchyma of an organ with drug [16, 17].

While there has been some work on the biodistribution and pharmacokinetics of ^99m^Tc-MIBI [18, 19], to the best of our knowledge, there has been no presentation of a gamma distribution convolution (GDC) model application in the nuclear or pharmacokinetic literature. GDC models have been applied elsewhere for ecological water storage I/O, waiting times in queueing theory, and in the evaluation of aggregate economic risk of portfolios [20]. Another possibility would be to construct a convolution model from observed input and organ functions using multiple ROIs as has been done for the kidney [16, 21, 22]. However, the advantages of finding a closed form convolution solution for TAC use, like the GDC model, are that only a single organ ROI is used, and that long-term data collection, as needed for washout-only modelling, becomes a shorter term requirement. Finally, unlike convolution methods using observed input functions, a closed form convolution is self-contained and thus useful as a surrogate for otherwise impossible to perform exhaustive testing while still maintaining a degree of realism.

In this context, we aim to demonstrate that following a peripheral intravenous bolus injection, the thyroid has a TAC that can be modelled accurately using the closed form convolution of two gamma distribution (GD) functions. The first GD of the GDC approximates thyroid gland marker arrival and distribution, and the second GD, the thyroid marker washout. We apply this model to TAC’s from dynamic ^99m^Tc-MIBI scans, and show how it can be used to calculate organ statistics, especially MRT values. Next, we perform a surrogate test example, Monte Carlo simulation of self-consistency of the GDC algorithm to check the accuracy of modelling. Finally, we compare the GDC, a more complete model of concentration, to four of the best available washout-only models for our 90-minute data.

## Theory

A convolution of two random variables can be used to form an impulse response model of time activity of an imaged organ’s radioactivity [14]. The first, fastest random variable is the impulse or blood pool that feeds the organ, and the second, slower random variable is the washout of activity from that organ. Note (1) that random variables are modelled as density functions and add by convolution, (2) that the total area of any density function is one, and (3) that any convolution of density functions is itself a density function with a total area of one [23, 24]. Closed form convolutions that model both arrival and washout have also long been used as pharmacokinetics models as originally inspired by Bateman’s treatment of radioactive density functions of parent (be^− bt^) and daughter species (*βe* ^− *βt*^) [25]. This latter is perhaps best written as the exponential density function convolution (EDC)

1 $$ \mathrm{E}\mathrm{D}\mathrm{C}\left(b,\beta; t\right)=\mathrm{b}{e}^{-bt} \otimes \beta {e}^{-\beta t} = \left\{\begin{array}{l}\left.\begin{array}{l}\mathrm{b}\beta \frac{e^{-\beta t}-{e}^{-bt}}{\mathrm{b}-\beta },\ \mathrm{b}\ne \beta \\ {}\kern1.75em {\mathrm{b}}^2t\ {e}^{-\mathrm{b}t}\kern0.5em ,\ \mathrm{b}=\beta \end{array}\right\}t\ge 0\\ {}\left.\kern3.75em 0\kern4.1em \right\}t<0\end{array}\right.. $$

The amount of daughter (or time activity) at time zero, i.e., the initial EDC functional height, is zero in this particular non-negative-valued Bateman equation. However, when taken out of the parent-daughter decay context, Eq. (1) becomes only approximate. Generally, whatever an exponential distribution (ED) can model is typically better modelled by a gamma distribution (GD) [26]. Exponential distributions are not the preferred shapes to explain organ bolus input function shapes, for which GDs or other functions are more useful [27–29]. To model the prolonged washout kinetics, which models are only fit following peak organ activity, GDs, one of several possible generalizations of EDs, have better fidelity than mono- and bi-exponential models [12, 13, 30–33]. One reason for this is that GD washout models imply zero initial drug volume with no initial mixing [33]. Thus, a GD convolution (GDC) should better fit an organ TAC than Eq. (1), as both the organ delivery and organ washout are more realistically modelled using GDs than EDs. The b = *β* solution to Bateman Eq. (1) can be written as a gamma density; GD(2, b; *t*) and its *n* ^th^ convolution as GD(*n*, b; *t*). However, b = *β* is statistically implausible, and *n* is only ever an integer, thus despite claims to the contrary [34], GDs do not derive from serial EDCs. Rather, Eq. (1) is a special case GD convolution–see the text following Eq. (5), and a derivation of the GD appears elsewhere [35].

The arrival of the ^99m^Tc-MIBI within the thyroid is not instantaneous. The fast signal or blood-pool activity within the thyroid is approximated here using a gamma distribution (GD_fast_) density function that for time *t* (min) is time-offset by *t*_Α_ ('A' for arrival) and zero before *t*_Α_ by letting *τ* = *t* − *t*_A_, and setting

2 $$ {\mathrm{GD}}_{\mathrm{fast}}\left(\mathrm{a}\kern0.2em ;\ \mathrm{b};\ \tau \right)=\left\{\begin{array}{cc}\hfill \frac{{\mathrm{b}}^{\mathrm{a}}}{\Gamma \left(\mathrm{a}\right)}{\tau}^{\mathrm{a}-1}{e}^{-\mathrm{b}\kern0.1em \tau },\hfill & \hfill \tau\ \ge\ 0\hfill \\ {}\hfill \kern2.75em 0\kern1.5em ,\hfill & \hfill \tau <0\hfill \end{array}\right., $$

where, if the dimensionless shape parameter a > 1 would yield a skewed bell curve and if a < 1 a decaying saw tooth or spike, and where b is the rate scalar (per min).

Washout functions are the impulse response to an instantaneously distributed signal and strictly washout. A washout function can be modelled as a gamma distribution,

3 $$ {\mathrm{GD}}_{\mathrm{WO}}\left(\alpha; \beta; \tau \right)=\left\{\begin{array}{cc}\hfill \frac{\beta^{\kern0.1em \alpha }}{\Gamma \left(\alpha \right)}{\tau}^{\alpha -1}{e}^{-\beta\;\tau },\hfill & \hfill \tau \ge 0\hfill \\ {}\hfill \kern3em 0\kern1.2em ,\hfill & \hfill \kern1.4em \tau <0\hfill \end{array}\right., $$

where *α*  < 1 is the shape parameter condition for washout, i.e., a monotonic decrease of functional height in time. Next, the GDC model is created by convolving GD_fast_ and GD_WO_,

4 $$ \mathrm{G}\mathrm{D}\mathrm{C}\left(\mathrm{a}\kern0.1em ,\mathrm{b},\alpha, \beta; \tau \right)={\mathrm{GD}}_{\mathrm{fast}}\left(\mathrm{a},\mathrm{b};\;\tau \right)\otimes {\mathrm{GD}}_{\mathrm{WO}}\left(\alpha, \beta; \tau \right), $$

Substitution of Eqs. (1) and (2) into Eq. (4) leads to

5 $$ \mathrm{G}\mathrm{D}\mathrm{C}\left(\mathrm{a}\kern0.1em ,\mathrm{b}\kern0.1em ,\alpha, \beta; \tau \right)=\left\{\begin{array}{cc}\hfill \frac{{\mathrm{b}}^{\mathrm{a}}{\beta}^{\alpha }}{\Gamma \left(\mathrm{a}+\alpha \right)}{e}^{-\mathrm{b}\tau }{\tau^{\mathrm{a}+\alpha}}^{-1}{}_1F_1\left[\alpha, \mathrm{a}+\alpha, \left(\mathrm{b}-\beta \right)\tau \right],\hfill & \hfill \kern.70em \tau >0\hfill \\ {}\hfill \kern3em 0\kern5.6em ,\hfill & \hfill \kern3.4em \tau \kern0.30em \le \kern0.30em 0\hfill \end{array}\right., $$

which is a density function consisting of a gamma variate multiplied by _1_ *F*_1_(A; B; Z), where the latter is a confluent hypergeometric function of the first kind^1^ [36, 37]. For a = 1 and *α* = 1, Eq. (5) reduces to Eq. (1), which in turn demonstrates that Eq. (5) is more general than Eq. (1). That is, in practice, the GDC would reduce to a Bateman density if a = 1 and *α* = 1 were plausible parameter values. For b = *β*, Eq. (5) reduces to GD(a + *α*, b; *τ*), a statistically implausible simplification used nonetheless [34]. Although serial GD convolution is known [20, 26], the easily recognizable simple closed form for the two GD convolution of Eq. (5) is a recent development [see [20], Eq. (2)].

Mean residence time, MRT, is independent of any TAC scaling, and its value can be calculated directly from a density function, *f*(*t*), as follows:

6 $$ \mathrm{M}\mathrm{R}\mathrm{T}={\displaystyle {\int}_0^{\infty }t\ f(t)dt}. $$

MRTs are additive [15]. That is, the mean residence time of the sum of random variables is the sum of the mean residence times of the random variables.

7 $$ {\mathrm{MRT}}_{\mathrm{GDC}}\to {t}_{\mathrm{A}}+\mathrm{M}\mathrm{R}{\mathrm{T}}_{\mathrm{fast}}+{\mathrm{MRT}}_{\mathrm{WO}}={t}_{\mathrm{A}}+\frac{\mathrm{a}}{\mathrm{b}}+\frac{\alpha }{\beta }. $$

However, tracer residence *within* the thyroid starts at the time of tracer first arrival within that organ, such that *t*_A_ is unrelated to thyroid residence time. Moreover, ${\mathrm{MRT}}_{\mathrm{fast}}=\frac{\mathrm{a}}{\mathrm{b}}$ quantifies the delivery and uptake of tracer in the thyroid, and only $\mathrm{M}\mathrm{R}{\mathrm{T}}_{\mathrm{WO}}=\frac{\alpha }{\beta }$ relates to how the thyroid washes out of tracer activity once that activity is distributed within the thyroid. That is, MRT_WO_ characterizes the response of thyroid tissue to an instantaneous signal.

## Methods

### Patient population

The data from three patients were acquired at the Hacettepe University Medical School and were processed blindly at the University of Saskatchewan. The ^99m^Tc dosages were 740 MBq. Patients 1 and 3 underwent ^99m^Tc-MIBI scanning for metastatic screening, and were negative. Patient 2 underwent parathyroid scanning for chemical hyperparathyroidism. On scanning, there was ^99m^Tc-MIBI retention in the lower part of the right thyroid lobe ROI found on histological examination to be a parathyroid adenoma measuring 11 × 11 mm. The GDC curve analysis was performed blindly and without knowledge of the region drawing, the scanned images, or the clinical presentation and results. Clinical correlation was performed only after the quantitative GDC results were obtained.

### Data processing

Following a peripheral intravenous bolus injection, 89 one-minute per frame dynamic ^99m^Tc-MIBI gamma camera images were obtained. Regions of interest (ROIs) were drawn over the right and left thyroid lobe in each frame, and the regional counts per minute were then used to construct time-activity curves (TACs). The TACs were then least Poisson noise model fit with the decay simulated GDC model, Eq. (5)—see the Appendix section—in a separate institution using Mathematica 10.3 programs using an algorithm stop condition of a 10^-20^ relative difference between successive iterations. Caution was taken to find global minima by including reasonable initial parameter range, starting values, but there is no absolute guarantee that global minima will be found with the Nelder-Mead (simplex) regression used. Poisson model noise calculations, regression fitting for Poisson models, corrected fit error quantification, and an algorithm for finding TAC starting-time values, *t*_A_, can be found in the Appendix section.

### Time-activity curve simulations

Monte Carlo simulations were performed for the purpose of estimating how accurate and precise the GDC results were. The Monte Carlo simulations of each case without decay correction were performed using each case’s GDC parameters as starting values to generate TACs. The TACs were then randomized ×10 by injecting pseudorandom Poisson noise and refit with GDC models using estimated Poisson loss functions to recover the altered (test) parameters. Unlike for the clinical data, the simulations had no injected misregistration. Radioactive decay was not simulated in order to (1) partly compensate for the lack of injected misregistration by increasing the noise to larger levels due to generating Poisson model noise from larger, not decay-diminished signals (2) crosscheck the accuracy of the decay-corrected GDC model used for the clinical cases, (3) simulate drug assay when decay is not a factor, and (4) provide an example of how simulations studies can be used to model variations of experimental conditions and validate techniques.

### Washout model analysis with comparison to GDC models

Comparisons of GDC MRT_WO_ results were made to four washout models; two different regression methods of fitting biexponentials, and two different regression methods of fitting single gamma variates. One of these latter, Tk-GV; the Tikhonov regularized gamma variate herein adaptively minimizes the relative error of *β* or alternatively the minimum relative error of plasma clearance [12, 13, 32]. Briefly, Tk-GV is an approximate inverse solution to Eq. (4). That is for *C*(*t*) = GD_fast_Tk-GV_WO_, then Tk-GV_WO_ = GD_fast_^(−1)^ ⊗ *C*(*t*), and Tk-GV fits an inverse (virtual) function, not concentration itself, *C*(*t*).

## Results

**Fig. 1:**
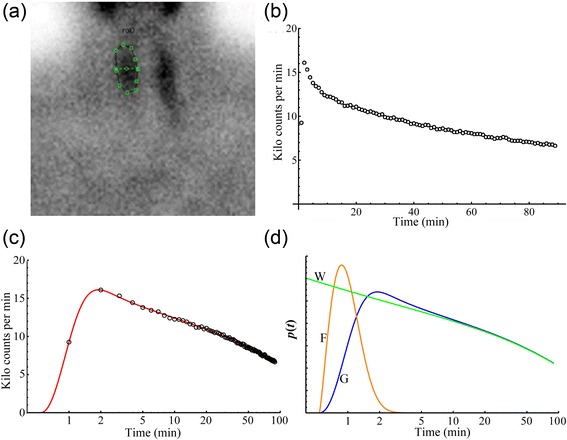
The *top left panel* (**a**), shows a region of interest (ROI) drawn over a right thyroid lobe of an example one-minute image from a ^99m^Tc-MIBI gamma camera image sequence. The *top right panel* (**b**) shows the ROIs time-activity curve (TAC). Panel (**c**) shows the same right thyroid lobe TAC as in panel (**b**) with time on a logarithmic scale to better display the rapidly changing curve at early times. Superimposed is the fit of a gamma distribution convolution (GDC, *solid red line*) scaled to the TAC showing a good fit to the TAC (*open circles*). Panel (**d**) shows graphical representations of the GD_fast_, the fast vascular gamma distribution (*F*, *orange);* GD_WO_, the washout gamma distribution (*W*, *green*) and the GDC; the convolution of GD_fast_ and GD_WO_ (*G*, *blue*). For clarity, the vertical axis has an arbitrary scale to superimpose the GD_fast_ function with the other two functions on one graph. Note that although the GD_WO_ eventually converges to the GDC model, this takes a long time to occur

A representative example of a ^99m^Tc-MIBI scintigram from a right thyroid lobe ROI is shown in Fig. 1a, while the corresponding TAC for the entire dynamic scan is shown in Fig. 1b. Figure 1c shows the same TAC with time on a logarithmic scale to display the rapidly changing curve at early times. Superimposed is the fit of the gamma distribution convolution (GDC) scaled (*S*) to the TAC. Figure 1d shows graphical representations of the fast vascular gamma distribution (GD_fast_), the washout gamma distribution (GD_WO_) and GDC; the convolution of GD_fast_ and GD_WO_.

**Fig. 2:**
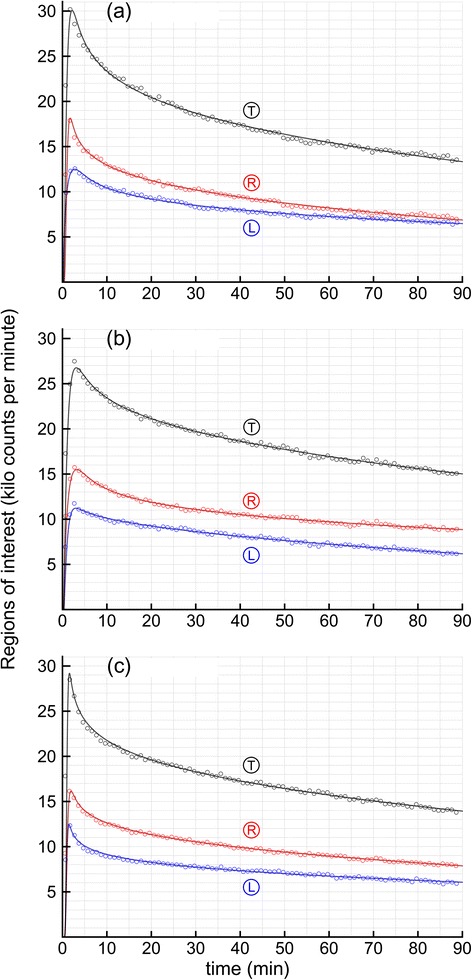
Shown below are unmodified data and decayed gamma distribution convolution fit results for three patients their left thyroid lobes Ⓛ, right thyroid lobes Ⓡ, and the total summed kilo counts per min for the whole thyroid glands Ⓣ. The *small circles* are the ROI counts in each one-minute frame for minutes 1 through 89. The *solid lines* are the GDC fits of the text. Note the quality of fit, as quantified below (as Fit err % in Table 1). **a** patient 1 **b** patient 2 **c** patient 3

Figure 2 shows the data and fits of the GDC for all nine TAC’s for the three patients. The parameter values obtained from fitting the GDC to the TACs and the GDC fit errors are summarized below in Table 1. The GDC parameters from Table 1 were used to calculate mean residence times (MRT) for the thyroid ROIs shown in Table 2. Note in Table 2 that the GD_fast_, a.k.a., thyroid vascular mixing times, the MRT_fast_ values, averaged only 0.807 min or 0.2% of the 367.4 min MRT_WO_, i.e., washout times. MRT_fast_ is the ratio of the 'a' and b values in Table 1, and is stable even though the shapes ('a' values) of the GD_fast_ distributions are quite variable due to the 'a' and b values being highly correlated (*r* = 0.99935). The MRT_WO_ values ranged from 211.6 to 699.0 min. Note that the left lobe, case 2L in Table 2, had the shortest MRT of only 211.6 min, whereas the same patient’s right lobe, 2R, had the longest MRT value, 699.0 min.

**Table 1:** Parameters for the GDC fit model

Pt thyroid^a^	*t*_A_ (min)	a	b (min^−1^)	*α*	*β* (min^−1^)	*S* (10^6^ cts)^b^	Fit err %^c^	Noise %	1 − R^2^ (%)^d^
1L	0.195	0.846	0.909	0.8577	0.001849	3.529	1.713	1.174	0.534
1R	0.334	3.733	5.211	0.8661	0.004081	2.319	1.914	1.094	0.350
1T	0.277	1.631	2.060	0.8645	0.003106	5.222	1.421	0.800	0.271
2L	0.340	1.351	1.692	0.9408	0.004445	2.027	1.690	1.183	0.630
2R	0.282	0.995	0.928	0.8668	0.001240	6.510	1.353	1.022	0.395
2T	0.307	1.127	1.203	0.8998	0.002652	6.625	1.019	0.773	0.239
3L	0.306	7.878	11.644	0.8918	0.002029	3.201	1.801	1.225	0.818
3R	0.425	3.082	4.520	0.8942	0.002863	3.296	1.026	1.060	0.189
3T	0.374	6.470	9.842	0.8947	0.002554	6.287	0.988	0.801	0.224

^a^Patient numbers 1,2,3 plus L left thyroid lobe, R right thyroid lobe, T total thyroid TACs, e.g., 1L, 2L

^b^ *S* is the scale factor used to equate AUC_TAC_ = *S* AUC_GDC_. The AUC of the GDC is one, as it is for all density functions. The AUC of a TAC is the total counts collected in the ROI from time is zero to infinity

^c^The fit error was increased to offset for the effect of using 6 fit parameters in the GDC model—see Eq. (10)

^d^From correlation of the TAC with the GDC model for the 89 one-minute sample times

**Table 2:** Decay-corrected MRT values in min for thyroid delivery and washout from 9 GDC models

Patient	MRT_fast_	MRT_WO_
Thyroid	Delivery	Washout
	a/b	*α/β*
1L	0.931	463.9
1R	0.716	212.2
1T	0.792	278.3
2L	0.798	211.6
2R	1.073	699.0
2T	0.937	339.3
3L	0.677	439.5
3R	0.682	312.4
3T	0.657	350.3

In addition to the variability of parameters due to divergent kinetics between clinical cases, there is a lesser amount parameter variability for each case, a.k.a., within case variability. In order to crosscheck the case-wise accuracy and precision of the fitting algorithm, Monte Carlo simulations of activity were performed using the observed clinical parameters values with injected simulated counting (Poisson) noise without modelling radioactive decay or misregistration. As a result, although there was an overlap of the fit errors for the simulations versus clinical data, the simulations and clinical data had borderline significantly different fit error (*p* = 0.036, from paired sample two-tailed *t* testing of Fisher transformed *r* values). Comparison of the clinical GDC parameters to the noise injected recovered parameters allowed for an independent (if simplistic) measure of accuracy and precision of recovery of the parameters as listed in Table 3. The recoveries of the start times for simulation, *t*_A_, averaged an insignificant 0.035% error (*p* = 0.98) suggesting that the start time algorithm of the Appendix section, Eq. (12), functioned adequately. The mean decay corrected thyroid washout MRT values went from 367.4 min from the 9 clinical regressions to 369.1 min from the average of 90 simulations for an absolute error of 0.46%. Note in Table 3 that none of the GDC simulation MRT values or other parameters (apart from the fit errors mentioned above) were significantly different from their corresponding clinical parameter values. Finally, *α*-shape values were never close to one in the 99 simulations and clinical cases (absolute range from 0.8528 to 0.9445), such that the use of ED_WO_ = *βe* ^− *βt*^ in the Bateman Eq. (1) above rather than GD_WO_ would introduce severe non-linear misregistration into the data fitting.

**Table 3:** Poisson noise simulations and accuracy of recovery of generating parameters following regression

Parameters	*t*_A_	a	b	*α*	*β*	*S* ^a^	MRT_WO_
Units	min	none	min^−1^	none	min^−1^	10^6^ counts	min
Clinical mean values	0.316	3.013	4.223	0.8863	0.00276	4.335	367.4
Simulation mean values^b^	0.316	3.314	4.588	0.8859	0.00275	4.342	369.1
Units	Percentage (%)						
Mean simulation CV error^c^	2.4	0.23	1.6	0.26	3.1	2.6	0.69
Absolute error in percent^d^	0.035	10.0	8.6	−0.038	−0.38	0.15	0.46
Units	Probability						
Probability of no difference^e^	0.98	0.44	0.52	0.32	0.38	0.73	0.51

^a^The scale factors *S*, used to scale GDC, are the total counts collected in the ROI from time is zero to infinity

^b^Each set of clinical parameters for 9 cases was used to generate 10 different noisy data sets. The simulation mean values are from all 90 simulations

^c^This is the mean value of 9 coefficients of variation (CV), where each CV is from 10 simulations

^d^Error is 100 times mean simulation minus clinical values divided by mean clinical value time

^e^No significant differences to the 0.05 level from two-tailed *t* tests for zero difference between 9 paired samples using mean values of 10 simulations for each clinical result and the clinical parameter results themselves

From the preceding, the 90 min of data may be sufficient for obtaining a complete concentration model, the GDC. Next, we tested to see if 90 min was a long enough time to obtain stable washout-only model MRT values. Table 4 shows a comparison of the 1st through 89th minute TAC GDC models with 5th through 89th minute TAC washout models. Two of the four 5th through 89th minute washout models are ordinary least square (OLS) models; biexponential OLS E2 and gamma variate OLS GV. The other two models are not concentration models. Those are 1/*C*(*t*)^2^ weighted least squares E2, and the Tk-GV model minimized for least relative error of *β*; WLS E2 and Tk-GV. For each method, how well the L and R MRT values produced interpolated total thyroid MRT values was calculated as a coefficient of variation as shown in Table 4. These CV(interpolation) varied from a high value of 51.9% for OLS E2 to a low value of 15.9% for GDC. Note that due to patient variation a CV(interpolation) of zero is not expected. The MRT_WO_ values for OLS E2 were of significantly shorter duration than those from the GDC model (Wilcoxon, *p* = 0.004).

**Table 4:**
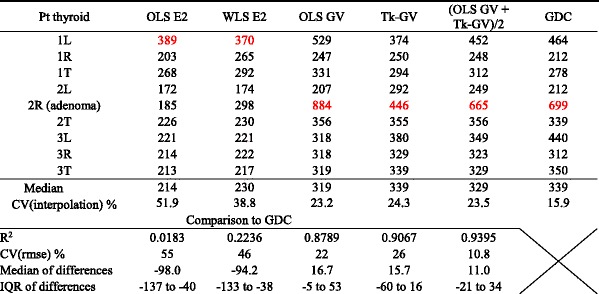
Mean residence time (MRT_WO_ in minutes) results for simple washout models fit from the 5th to 89th minute ROIs and compared to the gamma distribution convolution (GDC) MRT_WO_ of the text^a^

^a^Regressions used were ordinary least squares (OLS), weighted least squares [WLS; 1/*C*(*t*)^2^ weighting], and an inverse method; Tk-GV. These were applied to biexponential (E2) and gamma variate (GV) functions. The longest MRT value for each method is in *red*. IQR is interquartile range. How well the total (T) thyroid interpolated the L and R MRT values was calculated as a coefficient of variation of interpolation, CV(interpolation), from the standard deviation of the distances to interpolation, *d* = MRT_Total_ − min{MRT_L_, MRT_R_}, divided by the mean of their interpolation interval, *ii* = |MRT_L_ − MRT_R_|. The CV of the root mean square error CV(rmse) was calculated for method *M* ≠ MRT_GDC_ as ${\left\{{\displaystyle \sum_{i=1}^n\frac{{\left[\mathrm{M}\mathrm{R}{\mathrm{T}}_M(i)-\mathrm{M}\mathrm{R}{\mathrm{T}}_{\mathrm{GDC}}(i)\right]}^2}{n}}\right\}}^{\frac{1}{2}}{\left[{\displaystyle \sum_{i=1}^n\frac{\mathrm{MR}{\mathrm{T}}_{\mathrm{GDC}}(i)}{n}}\right]}^{-1}$. The median of differences was taken pair-wise. Note that the errors for OLS GV and Tk-GV appear to be, on average, in opposite directions

As can be seen in Table 4, the smallest and only statistically not significant R^2^ value (0.0183, *p* = 0.7) was between OLS E2 and GDC. The parathyroid adenoma (2R) was not identified by its MRT_WO_ using OLS E2 and WLS E2, but was the longest MRT_WO_ for all of the GV-based models and for the GDC model. Although the GV washout models; OLS GV, Tk-GV, and the average of OLS GV and Tk-GV were superior to E2 models for the single-case lesion detection, the highest R^2^ value with the GDC of 0.9395 for those washout models occurred for the average MRT_WO_ values of OLS GV and Tk-GV. This averaged model MRT_WO_ value corresponded to a 10.8% coefficient of variation of root mean square error, CV(rmse), agreement with GDC MRT_WO_. This compares to the Monte Carlo testing of the GDC MRT_WO_, with a 0.69% (CV) for precision and 0.46% for accuracy—see Table 3.

For the results listed, we processed the TACs as supplied. These neither represented the values for single thyroid lobes nor for whole thyroid glands but rather a mixture of those values. For GDC models of whole thyroid glands listed in Table 4, the IQR of MRT values was 72 min. The GDC IQR from the given mixture of regions was 206 min, and for thyroid lobes without whole thyroid MRT values, the IQR value was 311 min (via Excel PERCENTILE.EXC subtraction or equivalent algorithm).

## Discussion

The quality of the fit results illustrated in Fig. 2 and listed in Table 1 suggest that gamma distribution convolutions, GCDs, can be used to form scaled models that accurately follow TACs of ^99m^Tc-MIBI activity in the thyroid. A GDC implies two gamma distributions, GDs: a GD_fast_ delivery to the thyroid function and a slower GD_WO_ washout from the thyroid function. Note that these separate gamma distributions were not observed directly, but rather were implied by the thyroid ROI data and the GDC fitting parameters. To illustrate these behaviours, the individual GD_fast_, GD_WO_, and GDC density functions are displayed in Fig. 1d. That figure shows that the effect of the GD_fast_ on the GDC dissipated in time, so that eventually the GD_WO_ curve converges to the path of the data (after ~1 hr). This, and the results in Table 4, confirm that washout-only models are only indirectly related to TAC or concentration models [14, 15], and that, in turn explains why washout models appear to require more extended time-sampling than the GDC model. For example, the GDC residuals were homoscedastic over the entire sampling space, which is never the case for washout-only models as washout models are at a maximum value initially, when actual concentration is zero. It has been shown in other systems that washout models, even with a 10 min start time, statistically significantly mismatched early data and agreed better with late data [12].

Most pharmacokinetic models are thought of as successful when their curve fit errors average 10% or less [as rmse in 38]. More important than curve fitting error is how precise the models are for the parameter of interest, or in our case, MRT_WO_ *.* We note that no washout model for predicting GDC MRT_WO_ had less than a 10% precision error measured as CV(rmse)—see Table 4—to unequivocally suggest its use with only 90 min of ^99m^Tc-MIBI data. The average MRT_WO_ for OLS GV and Tk-GV correlated best to GDC MRT_WO_. The OLS GV is a direct fit to concentration and Tk-GV is an inverse solution, virtual concentration fit, and which methods have errors in opposite directions when applied to only 90 min of data. This combined MRT measurement had an error of 10.8% compared to the GDC model, i.e., it was almost good enough for clinical use. Thus, the GDC model may find use as a standard for investigating simpler washout models. Herein, each OLS model (OLS E2 and OLS GV) was outperformed for MRT_WO_ R^2^ with GDC by its corresponding weighted alternative; WLS E2 and Tk-GV. Alone amongst the methods tested, MRT_WO_ OLS E2 did not (statistically significantly) regress or correlate to MRT_WO_ GDC (or to Tk-GV). Better correlated was WLS E2. The 1/*C*(*t*)^2^ least squares weighting fits biexponential washout models favour fitting the later data to obtain more accurate half-lives [39]. Although some researchers have, without much discussion, used WLS E2 rather than OLS E2 in their patient series [13, 40, 41], direct numerical evidence of improved correlation against better or more complete models, such as that in Table 4, is currently limited. From the Monte Carlo testing—see Table 3—the GDC MRT_WO_ had a 0.69% (CV) precision and 0.46% accuracy, so that comparison to GDC as a measure is not obviously error prone.

No area normalized background subtraction using a second ROI outside of the thyroid ROI was performed for our analysis. For the current work, background was considered to be the blood pooling within the thyroid region as modelled by the fast or delivery function activity, whose short time-duration (MRT_fast_ in Table 2) agrees in a general way with the short time that ^99m^Tc-MIBI is known to remain in the blood [11]. The background adjustment herein was to treat the blood pooling in the thyroid ROI as the delivery vehicle to the extravascular tissue within the same ROI for later washout. This is similar to the fast decaying quasi-exponential functional treatment of background for ^99m^Tc-MAG3 renal scintigraphy by the recently developed blood-pool compensation and modified Patlak-Rutland methods both of which do not use classical background regions [17, 42–44]. The shape of the fast curve, GD_fast_, i.e., the individual 'a' values in Table 1 were variable and implied shapes sometimes heavier tailed than exponential distributions (a < 1), and sometimes lighter-tailed, i.e., closer to normal distributions (a > 1). It would be remiss to imply that the GD_fast_ curve shape is accurately captured, i.e., the GD_fast_ shape coefficient, 'a' was quite variable—see Table 1. The difficulty in quantifying the GD_fast_ shape is likely due to the unavoidable sparseness of nuclear decay data. Using faster framing rates to capture the shape of the fast function better would likely increase both misregistration and noise as these measures covaried (*r* = 0.77 see *Misregistration* in the Appendix section). However, the mean MRT_fast_ times from patient studies, numerically a/b, were quite stable as the 'a' and b values were highly correlated (*r* = 0.99934). From the simulations, the mean standard deviation MRT_fast_ time was only 1.0 s (within cases), where the population standard deviation (between cases) was 0.137 min (8.2 s) suggesting that two parameter GD_fast_ functions are adequate for calculating MRT_fast_ values. The washout function, GD_WO_, has a shape parameter, *α* < 1, which yields a curve that is monotonically decreasing (washing out), with very stable shapes herein [CV(*α*) = 2.9% from patient studies, 2.6% from simulations between cases, and 0.26% from simulations within cases]. The rate scalar (*β*) of the GD_WO_ is much smaller (i.e., slower) than the rate scalar (b) of the fast curve. Combined with concentration curves, washout models are used for predicting clearance, as they conserve mass. However, washout models do not form accurate models of early time TACs—see Fig. 1d—and [45], and that is a good motive for creating more complete models, i.e., that better fit the data, with the alternatives being (1) to ignore the early data, extend the data collection for hours, and to use models whose misregistration magnifies substantially outside of fit range—see [12, 45], or (2) include the early data and suffer significant, large misregistration within the fit range—see [12]. Despite the pleomorphic shapes of an assumed fast function, and the noise problem, the resulting GDC functions’ misregistration of the TACs counts was only 0.80% with a total fit error of 1.44% (see *Misregistration* in the Appendix section). Using our threshold measure of goodness-of-fit for curve fit errors of 10%, the GDC TAC error was an order of magnitude better fitting than most models [38].

MRT values were obtained here without performing absolute uptake calculations, drawing more than one ROI per thyroid lobe, or acquiring data for a long time, e.g., 6 h, see [18]. The GDCs models’ decay corrected mean residence times (MRT_WO_) of ^99m^Tc-MIBI in the thyroid TACs ranged from 211.6 to 699.0 min. However, not all of our results were from region drawing over normal tissue. We observed considerable differences between the left and right thyroid lobe MRTs, even in our sample of only three cases. Patients 1 and 3 had neoplastic disease elsewhere and no thyroidal metastatic disease detected on scanning. However, patient 2 (2R) had an inferior pole of right lobe of thyroid region 11 × 11 mm parathyroid adenoma. In diseases such as hyperparathyroidism and parathyroid adenoma/carcinoma, thyroid metabolism is presumed normal while the parathyroid tissue is abnormal and typically hyperplastic hypometabolic (containing enlarged, slow to washout parathyroid glands). Indeed, the MRT for that entire right lobe was the slowest in this series at 699 min even though the adenoma was much smaller than the whole ROI. When the patient with thyroid pathology was excluded, the two remaining total thyroid MRTs had values of 278 and 350 min, or not dissimilar to the published thyroid MRT of 314 min obtained using a monoexponential washout model and 6 h of imaging data [18]. This blinded study may have unintentionally detected an adenoma using the GV models. All of the GV models had their longest values associated with the parathyroid adenoma containing thyroid lobe TAC. Usually far outliers (>3 IQR) should receive some comment, and this was the case for OLS GV for the parathyroid adenoma containing thyroid lobe. That GDCs MRTs are proper measurements is evidenced by (1) the adenoma lobe’s MRT being the longest GDC-MRT time, (2) having lowest total (T) thyroid MRT value interpolation error between L and R thyroid lobes,—see Table 4—and (3) the very low misregistration of the GDC model. However, as the interpatient variation of hot spot MRT values is unknown *this case series is far too small* to the established clinical utility for any MRT calculation. We also perceive a need to establish robustness of the GDC fit algorithm in the clinical setting and to establish any potential applicability to myriad other thyroid pathologies for which the visual clues on scanning are not as obvious as for hot spot imaging. On the other hand, Table 4 shows that the biexponential, OLS E2 and WLS E2, values (1) failed to identify the adenoma containing thyroid lobe, (2) had poor quality interpolation results of L and R thyroid lobe MRTs, and (3) had large modelling errors. Thus, *this case series was large enough* to demonstrate that the OLS E2 and WLS E2 models can be problematic for identifying hot spots with only 90 min of data. Our hypothetical explanation for this failure is that the MRT_WO_ values were significantly, spuriously, and similarly shortened due to the instant-mixing property of biexponential models. Instant-mixing range-restricts quantifying redistribution, and biexponential models borrow from the physical redistribution to make inflated mass elimination estimates [33]. Moreover, redistribution is the predominate cause of concentration dilution at early times following bolus delivery [33]. We hypothesize that physically, redistribution is generalized and similar for L, R, and T and that redistribution contamination makes the biexponential MRT values too fast and too similar, and thereby not properly interpolative and consequently non-diagnostic.

In prior works, the kinetic behaviour of ^99m^Tc-MIBI in the thyroid and parathyroid glands has been extensively explored using semi-quantitative parameters or visual examination of images acquired at different times [8–10]. Although severely limited by the retrospective nature of the current study, the GDC model should nonetheless allow for a prospective precise quantitative assessment of disease states using only 90 min of data for pharmacokinetic parameters such as the MRT, or at very least provides a model of concentration/counting for simulation studies for the evaluation of washout models of MRT. *This case series was large enough* to demonstrate that GDC functions can be of sufficiently high fidelity of fit to imply the use of GDC functions as templates for pharmacokinetic simulation studies, and in that capacity one can consider the results as demonstrating a proof of principle for simulation studies. Indeed, we checked the self-consistency of the clinical decay correction algorithm by comparing its results to the results from simulated data without drug decay.

For washout-only models, one should wait for at least several minutes before starting the fit of polyexponential washout models, and hours before fitting monoexponential washout models, and the fitted data should be collected for as many hours as possible. One alternative to such a fitting strategy is not to attempt to fit concentration itself, but to fit a different regression target, for example, minimum relative error of the rate scalar (*β*), or weighting toward the later time samples. However, we did not have enough elapsed time for sampling to do this accurately—see the Results section and Table 4. Our other strategy was to use the GDC; a more complete model from the addition of random variables, where chained random variables add by convolution. The use of convolution to create concentration models inclusive of early and late data is hardly new [15]. However, this has usually been done using sums of exponential terms for the washout models, which latter are less accurate and precise washout models than gamma distribution models see Table 4 and [13, 31, 46]. The current work is possibly a first application of a GDC radioactivity (or concentration) model. Many drugs follow GD washout curves [31]. GD washout models extrapolate to late time better than biexponential functions [12], and in their adaptively obtained form from Tikhonov regularization, as Tk-GV, are less affected by altered body fluid states than exponential models [32, 47, 48]. Thus, GDC model simulations with altered count rates or injected noise levels could be used to inspect the effects of altered ^99m^Tc-MIBI dosage, ROI size, framing rates, or start times for fitting models and regression targets including those for various washout functions and the GDC models themselves.

The Table 3 summary of Monte Carlo noise simulations illustrates the parameter accuracy one can expect from GDC modelling. The Appendix section Eq. (12) was used to estimate start times and appears to have functioned properly with very little error on simulation. The GD_fast_ parameters 'a' and b were somewhat inaccurately recovered following simulation. However, the most important MRT_WO_ results proved to be both accurate (0.46% mean error) and precise (mean CV 0.69%). An accurately determined parameter was *α* with a within case accuracy error of −0.038%. This parameter determines the shape of the washout curve, which is the predominant shape determinant of the GDC model. This explains the relative superiority of GD washout models to sums of exponential terms, as the latter have a rigid assumed shape and lack a shape parameter. Thus, it should also be possible to use the GDC model to test for the optimal time to first fit a washout model to a TAC, which given the disagreement between early activity and the typical washout model functional height, is generally thought to be ≥ 5 min for gamma variate washout model adaptive fitting or polyexponential model fitting [12, 13, 32] and > 1 h to 6 h, for monoexponential washout model fitting, which latter cannot be directly compared to the time limited gamma camera data herein [18]. In summary, what sample times are needed for fitting a washout model to data so that the parameters obtained are accurate has not been systematically examined, but the methods herein provide a model that should be useable for that purpose.

One of the limitations of the current retrospective study was that the gamma camera acquisitions in the parent series were started after tracer first arrival in the thyroid. However, for the studies processed and for our simulations, a minor delay in the acquisition start time proved to be somewhat forgiving—see the Appendix section for details. Nevertheless, further work on calculating the time of first arrival of activity in the target organ and examination of a larger patient series may be useful. The GDC model was processed using 90 min of data, and although the GDC model MRT_WO_ results appeared to be meaningful thyroid/parathyroid measurements, the GDC model as applied to longer imaging times remains untested. Thus, the conclusions are preliminary.

## Conclusions

By using thyroid ^99m^Tc-MIBI scintigraphy data from 3 patients, we generated 9 TACs and 90 Monte Carlo TAC simulations using gamma distribution convolution (GDC) models. The GDC models fit the TAC data with high fidelity and small enough TAC noise-image misregistration (0.8%) that they have a plausible use as simulations of thyroid activity for querying performance of other models, such as washout models, for altered ROI size, noise, administered dose and image framing rates. Monte Carlo accuracy testing results for all of the GDC model parameter values were good with a GDC MRT accuracy of 0.46%, despite fitting only 90 min of data. Since the GDC model is an actual concentration model, it does not have to decay several half-lives in order to obtain model parameters or validate the methodology. The 4 washout-only models applied to the same data all exceeded 10% precision error compared to the GDC, with the apparent excess error suspected to be from having insufficient temporal data for washout modelling.

## Appendix

Expected Poisson noise calculation, fitting to minimize the noise of counting, and calculation of fitting error

The expected (Poisson model) noise (*N,* noise percent, Table 1) from counting increases as the square root of the number of counts per frame in the ROI, which as a percentage for the *n* frames of the TAC is calculated from its root mean square (RMS) value

8 $$ N=\frac{100}{n}{\displaystyle \sum_{i=1}^n\frac{1}{\sqrt{\mathrm{TAC}\left({t}_i\right)}}}\ . $$

To scale amplitude of the GDC to the AUC of a TAC, a scale factor *S* (total counts collected in the ROI from time is zero to infinity, here) is used during the fitting process. Due to decay, the AUC of total counts is not an AUC of concentration. The fitting parameters were found by minimizing a norm of fit error for modelled counting noise

9 $$ {\mathrm{Min}}_{\mathrm{fit}}= \min \left\Vert \sqrt{\mathrm{TAC}\left({t}_i\right)}-S\ {e}^{-\lambda\ {t}_i}\frac{\mathrm{GDC}\left({t}_i-{t}_A\right)}{\sqrt{\mathrm{TAC}\left({t}_i\right)}}\right\Vert, $$

where *i* = 1 − 89 represent each of the 89 one-minute time samples, and the ^99m^Tc decay rate [49] is *λ* = ln(2)/[60(min/h) ⋅ 6.0067 (h)] ≈ 0.00192326. Decay correction using was performed on the fitted model in order to preserve the noise characteristics of the observed data during regression analysis. To express Eq. (9) as a goodness-of-fit containing error that includes that from noise, modelling error of fitting, gamma camera nonlinearity, and patient motion, one computes

10 $$ F=100\ \frac{n}{\sqrt{n-m}\ {\displaystyle {\sum}_{i=1}^n\sqrt{\mathrm{TAC}\left({t}_i\right)}}}{\mathrm{Min}}_{\mathrm{fit}}, $$

where *F*, the fit error percent in Table 1, from minimization has been augmented to correct for the *m* = 6 parameters of the GDC model (five GDC variables and one curve matching start time) by multiplying by $\sqrt{n}/\sqrt{n-m}$ and collecting terms.

Misregistration

Assuming counting error of a Poisson noise type, misregistration, an imaging term is defined graphically as the standard deviation vertical misalignment on a square root of count rate TAC plot, which latter is, indeed, an image. Misregistration standard deviation, *σ*_*M*_ = 0.79943%, was calculated from the well-known equation for correlated variances

11 $$ {\sigma_M}^2={\sigma_{F,N}}^2={\sigma_F}^2-{\sigma_N}^2-2{\rho}_{F,N}{\sigma}_F{\sigma}_N, $$

where the variance of misregistration, *σ*_*M*_^2^, is *σ*_*F*,*N*_^2^, the variance of the difference between fit error, *σ*_*F*_ = 1.44164% [from the standard deviation from Table 1 of Eq. (10)], and noise error, *σ*_*N*_ = 1.02864% [Eq. (8)], where *ρ*_*F*,*N*_ = 0.76531 is the correlation coefficient of *F* and *N*.

Calculation of a stable starting times (*t*_*A*_) for gamma distribution convolutions

The fit parameters *a*, *b*, and *t*_A_ are interrelated making a grid search for *t*_A_ poorly behaved. In practice,*t*_A_ starting values even a fraction of a minute away from the correct value can convert the GD_fast_ into a degenerate normal distribution (ND) of the type $\underset{\mathrm{a}\to \infty }{ \lim}\mathrm{G}\mathrm{D}=\mathrm{N}\mathrm{D}$ with either *t*_A_ → − ∞ or b → ∞. This occurs because as the shape parameter 'a' of the fast gamma distribution of Eq. (2) increases, the GD_fast_ mean time increases faster than its standard deviation, i.e., $\mathrm{f}\mathrm{o}\mathrm{r}\ \mathrm{a}>1,\ \mathrm{a}/\mathrm{b}>\sqrt{\mathrm{a}}/\mathrm{b}$, and that produces either a shift of the GD_fast_ curve to later time, or a large negative curve fit *t*_A_ start time (an ND starts at *t* = − ∞ and not at *t* = 0). To avoid this instability, an independent method of calculating start times was used. Herein we borrowed an algorithm from prior work, which suggested that the start time of a rapidly changing in time aortic signal can be calculated from back extrapolation to the activity is zero axis using the maximum slope of the aortic signal’s 0 to *t* integral (or more accurately its cumulative 0 to *t*_*i*_ sum) [16, 21]. This is because such an integral is monotonically increasing, which approximately linearizes its early slope, which slope is at a maximum value at very early times when the activity in the ROI is non-trivial. Using the first two available samples of the thyroid cumulative sum allow for the calculation of a start time from

12 $$ m=\frac{\left(y2+y1\right)-y1}{t2-t1}\to y-y1=\frac{y2}{t2-t1}\left({t}_{\mathrm{A}}-t1\right)\to {t}_{\mathrm{A}}=t1-y1\frac{t2-t1}{y2}, $$

which requires that *y*1 be non-trivial; it must be selected after *t*_A_ occurs, and that *y*2 > *y*1, that is, *y*1 should occur before peak organ activity. For an investigation of the Eq. (12) algorithm’s precision and accuracy see the Results section Table 3.

Note that the *t*_A_ values of Table 1 are framing rate offset. That is, the cumulative counts during the first minute are nominally assigned an occurrence time of 1 min, whereas the physical time of occurrence for a linear rate of change of counting during that minute would occur ½ min sooner. That means that the physical start times occurred at *t*_A_ − 0.5 min, and the gamma camera acquisitions were begun approximately 11 s after tracer first appearance in the thyroid. Whereas this guaranteed *y*1 values to be non-trivial, as required for use of Eq. (12), the second condition for use of that equation, that *y*1 should occur before peak organ activity, would be problematic for a delayed gamma camera start time late by much longer than a few seconds.

## Footnotes

1 The fast computation of this relies on ${}_1\mathrm{F}_1\left(\mathrm{A};\mathrm{B};\mathrm{Z}\right)=\frac{\Gamma \left(\mathrm{B}\right)}{\Gamma \left(\mathrm{B}-\mathrm{A}\right)\Gamma \left(\mathrm{A}\right)}{\displaystyle {\int}_0^1{\mathrm{e}}^{\mathrm{Z}\;t}{u}^{\mathrm{A}-1}{\left(1-u\right)}^{\mathrm{B}-\mathrm{A}-1}\mathrm{d}u}$, where A, B, and Z are variables and where the function is finite for all finite values of those variables. Also, we set GDC(⋅⋅⋅⋅; *τ* = 0) = 0 to replace the 0^0^indeterminacy of *τ* ^a + *α*− 1^ at a + *α* = 1, *τ* = 0 with its limiting value of 0.
